# Epileptic Phenotype and Cannabidiol Efficacy in a Williams–Beuren Syndrome Patient With Atypical Deletion: A Case Report

**DOI:** 10.3389/fneur.2021.659543

**Published:** 2021-06-08

**Authors:** Antonio G. Nicotera, Maria Spanò, Alice Decio, Giulia Valentini, Maria Saia, Gabriella Di Rosa

**Affiliations:** ^1^Division of Child Neurology and Psychiatry, Department of the Adult and Developmental Age Human Pathology, University of Messina, Messina, Italy; ^2^Neuropsychiatry and Neurorehabilitation Unit, Scientific Institute Istituto di Ricovero e Cura a Carattere Scientifico (IRCCS) Eugenio Medea, Bosisio Parini, Lecco, Italy

**Keywords:** antiepileptic drugs, cannabidiol, neurogenetics, pharmacoresistant epilepsy, Williams-Beuren syndrome

## Abstract

Epilepsy is a rare clinical manifestation in Williams–Beuren syndrome patients. However, some studies report the presence of infantile spasms and epilepsy in patients carrying larger deletions. Herein, we describe a 13-year-old female affected by Williams–Beuren syndrome and pharmacoresistant epilepsy reporting a *de novo* large heterozygous 7q11.21q21 deletion (19.4 Mb) also including the *YWHAG* gene. Studies indicate that cannabidiol is effective as adjunctive therapy for seizures associated with tuberous sclerosis complex, and it is under investigation also in focal cortical dysplasia. When treated with cannabidiol, our patient showed a significant reduction in seizure frequency and intensity, and improved motor and social skills. We hypothesized that CBD could exert a gene/disease-specific effect.

## Introduction

Williams–Beuren syndrome (WBS) is a rare genetic multisystemic neurodevelopmental disorder characterized by typical facial dysmorphisms, short stature, congenital cardiac defects, weakness of connective tissue, and mild-to-moderate intellectual disability (ID); it is caused by a 7q11.23 heterozygous deletion of 1.5–1.8 Mb involving 28 genes (1); however, larger atypical deletions are described in 5% of cases (2). Although epilepsy is rarely associated with common WBS deletions, some studies report the presence of infantile spasms and epilepsy in patients carrying larger deletions (2). Herein, we describe a 13-year-old female affected by WBS and report a *de novo* large heterozygous 7q11.21q21 deletion (19.4 Mb), also including the genes membrane-associated guanylate kinase WW and PDZ domain-containing 2 (*MAGI2*), Huntingtin-interacting protein 1 (*HIP1*), and tyrosine 3-monooxygenase/tryptophan 5-monooxygenase activation protein gamma (*YWHAG*). These three genes have been suggested as causative of epilepsy in patients with WBS and atypical deletions (3, 4). The patient showed a strong epileptic phenotype suggestive of pharmacoresistant epilepsy. In this regard, cannabidiol (CBD) has been recently approved by the United States Food and Drug Administration (FDA) as an adjunctive treatment of seizures associated with Dravet syndrome and Lennox–Gastaut syndrome (LGS). Moreover, preliminary data from a recently completed placebo-controlled trial indicate that CBD is effective as adjunctive therapy of seizures associated with tuberous sclerosis complex (TSC) (5, 6). Thus, we hypothesized that it could be helpful in treating pharmacoresistant epilepsy in our patient. Herein, we reported the remarkable beneficial effect of cannabinoids in treating seizures and speculated about the CBD action mechanism.

## Case Report

**Figure 1:**
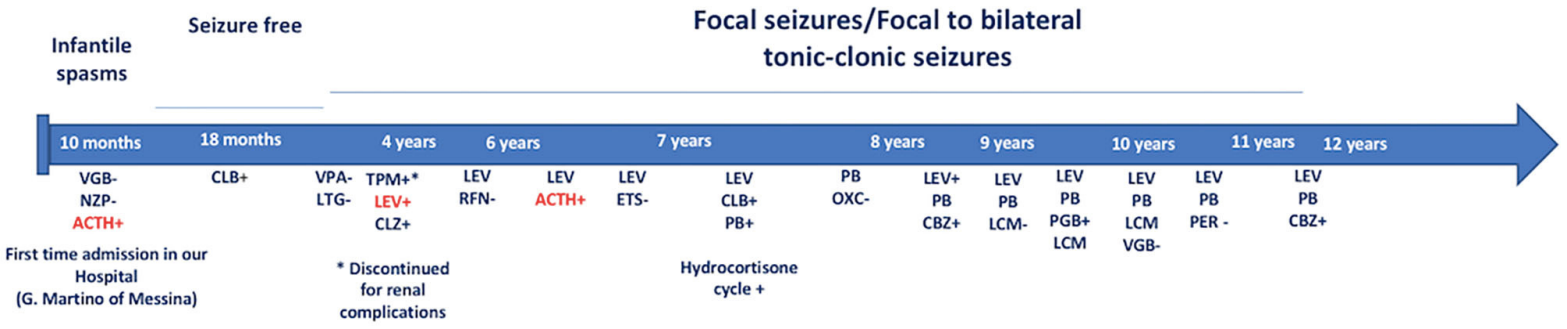
Onset of infantile spasms at the age of 10 months. She was treated with NZP and VGB without benefit. IM ACTH treatment and successively, CLB in monotherapy provided a seizure-free period. At the age of 2 years, the patient showed focal seizures/focal to bilateral tonic-clonic seizures that failed several AED's treatment. Due to the high frequency of the seizures (>4 attacks/day) and frequency episodes of epileptic the child has been monthly hospitalized. Until now, LEV and cycles of ACTH provided the more significant benefits. VGB, Vigabatrin; NZP, Nitrazepam; ACTH, adrenocorticotropic hormone; CLB, Clobazam; VPA, Sodium Valproate Acid; LTG, Lamotrigine; TPM, Topiramate; LEV, Levetiracetam; CLZ, Clonazepam; RFN, Rufinamide; ETS, Etosuccimide; PB, Phenobarbital; OXC, Oxcarbazepine; PGB, Pregabalin; LCM, Lacosamide; PER: Perampanel; CBZ, Carbamazepine. +: Good therapeutic efficacy (decreasing frequency and/or intensity of seizures). –: Bad therapeutic efficacy (increasing and/or worsening of seizure frequency or severity). In red: excellent therapeutic efficacy (marked decrease in frequency and/or intensity of seizures).

**Figure 2:**
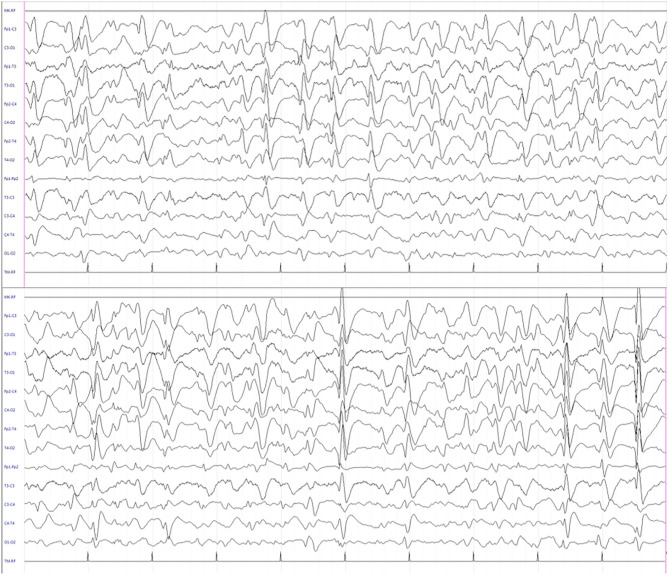
Interictal electroencephalogram during sleep showing quasi-continuous, centro-temporal, and high voltage spike-and-wave complexes, frequently followed by theta-delta activity.

**Figure 3:**
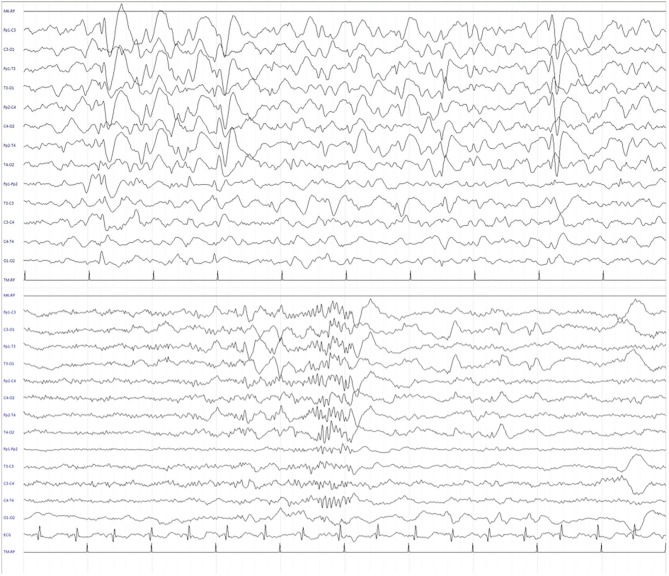
Electroencephalogram (EEG) during sleep after 3 months of CBD treatment. The recording showed a significant reduction of EEG abnormalities in frequency and amplitude, and the presence of sleep figures, including sleep spindles.

We report the case of a 13-year-old girl born from not consanguineous parents. She was born preterm (32 weeks of gestational age) after a pregnancy complicated by threatened miscarriage. The patient showed microcephaly, facial dysmorphisms (depressed nasal bridge, broad nose, wide and prominent open mouth, puffiness around eyes and lips, and full cheeks), failure to thrive, recurrent kidney stones, and scoliosis. A severe developmental delay became evident since birth. Neurologically, she reported flaccid tetraparesis without achievement of deambulation. Language skills were merely characterized by vocalizations. An echocardiogram revealed peripheral pulmonary artery stenosis. She was hospitalized for severe respiratory distress and neonatal seizures at birth time, requiring treatment with phenobarbital. At 10 months of age, she showed infantile spasms and, subsequently, the seizures presented high variability in intensity and semeiology, suggesting a diagnosis of drug-resistant epileptic encephalopathy. Multiple-label and off-label antiepileptic drugs (AEDs) were prescribed without clinical effects (Figure 1). At 2 years of age, a normal female karyotype was confirmed by proper testing, while fluorescent *in situ* hybridization (FISH) revealed typical WBS deletion del (7) (q21q21), confirming WBS diagnosis. A first brain magnetic resonance imaging (MRI) was obtained on the first day of life, but it was not informative. A new MRI was performed at the age of 11 years and revealed a thinning of the corpus callosum, reduced brainstem size, thinning of periventricular white matter, and bilateral optic nerve hypoplasia. At the age of 12 years, Agilent Human Genome CGH Microarray Kit 4 × 180K (with an overall median probe space of 13 kb) revealed a *de novo* very large deletion [del 7q11.21q21.11 (66849415_86269865) × 1 of 19.4 Mb]. Subsequently, we performed whole-exome sequencing (WES). The patient and her parents DNA were analyzed by OneSeq protocol, *via* Illumina HiSeq 2500, but any mutation in known genes associated with epileptic encephalopathy was reported. At the age of 13 years, the patient was readmitted to our Child Neurology Unit at A.O.U. “G. Martino” Hospital, Messina, because of a marked increase in frequency and severity of seizures (four to five seizures/day), with variable semiology (focal seizures or flexion–extension spasms mostly associated with cyanosis, bradycardia, and desaturation) and duration (from few seconds up to 20 min), often requiring treatment with endorectal diazepam and/or hydrocortisone. She was treated with primidone, carbamazepine, diazepam, and levetiracetam (LEV). Interictal electroencephalogram (EEG) showed *quasi-continuous* spike-and-wave complex in the frontocentral region (Figure 2). Primidone, diazepam, and carbamazepine were progressively reduced and discontinued. LEV was titrated up to 1,200 mg/day (63 mg/kg), pregabalin up to 50 mg/die (2.6 mg/kg); clobazam 20 mg/die (1 mg/kg) was added but without clinical benefit. On the 20th day of admission, the patient was started on Epidiolex—a new, 99% pure, oral CBD extract (Epidiolex, GW Pharmaceuticals, London, UK) −50 mg twice daily, then titrated up to 200 mg twice daily (20 mg/kg). After 3 months of CBD treatment, seizure frequency was reduced up to one to three attacks/week and characterized by sporadic and brief clusters of flexor-extensor spasms. No episode of cyanosis, bradycardia, and desaturation was reported. The patient did not need acute diazepam or hydrocortisone treatment. Moreover, an evident improvement of patient awareness and social interaction were noticed. An enriched vocalization and a gain of better postural and gross motor skills (reaching of sitting or crawling postures) were also noted. A new EEG was performed revealing an improvement in cerebral activity and a mild reduction of EEG abnormalities in comparison with her previous EEG (Figure 3). Specifically, the following findings were recorded: (i) theta-alpha activity was more frequently detected in awake; (ii) sleep spindles and K-waves were mainly noted in sleep compared to the previous registration; and (iii) the frequency of EEG abnormalities was reduced by 50 and 20% during sleep and awake, respectively. We scheduled a successive control visit after a 6-month CBD treatment; however, unexpectedly, she died at the end of June in another hospital due to complicated pneumonia. The patient's parents did not refer changes in her neurological clinical picture compared to the last valuation.

## Discussion

Epilepsy is a rare clinical manifestation in WBS patients. However, some studies report the presence of infantile spasms and epilepsy in patients carrying larger deletions (2, 3). We described a patient with WBS and pharmacoresistant epilepsy due to an atypical larger deletion (with the involvement of *MAGI2, HIP1*, and *YWHAG*, which are candidates as epilepsy genes). When treated with CBD, our patient showed a significant reduction of seizure frequency and intensity and improved motor and social skills. Thus, we hypothesized that CBD could exert a gene/disease-specific effect. Although the mechanism of epileptogenesis cannot be established with certainty in our patient, we hypothesized that *YWHAG* gene could explain the effects of CBD.

CBD may be an efficacious long-term treatment option in patients with pharmacoresistant epilepsy and received FDA approval for the treatment of seizures associated with Dravet syndrome and LGS in patients older than 2 years of age. Moreover, preliminary data from a recently completed placebo-controlled trial indicate that CBD is effective as adjunctive therapy of seizures associated with TSC; it is also under investigation in focal cortical dysplasia (FCD) (5, 8). These findings suggested an involvement of PI3K/Akt/mammalian target of rapamycin complex 1 (mTORC1) signaling pathway. mTORC1 is causally involved in a subset of malformations of cortical development in FCD and TSC; mTORC1 acts as a downstream effector for severely mutated pathways, including the PI3K/Akt and MAPK pathways. García-Rincón et al. revealed a striking increase in cannabinoid type 1 (CB1) receptor expression levels in FCD Type II and hypothesized that this enrichment occurs in neurons with overactive mTORC1 signaling (8). Currently, although the underlying mechanisms causing the anti-seizure effects of CBD remain unclear, available data indicate that it exerts multiple actions such as desensitization of transient receptor potential of vanilloid type 1 (TRPV1) channels, antagonism of G protein-coupled receptor 55 (GPR55), inhibition of adenosine reuptake, and a negative allosteric effect on CB1 receptors (5).

The *YWHAG* gene encodes for 14-3-3γ, a member of the 14-3-3 protein family which plays an important role in cell cycle progression and cortical development. 14-3-3γ provides a link between RAF-1 protein (involved in Ras/Raf/Mek/Erk (MAPK) pathway) and phosphokinase C, acting as a signal transduction protein regulated by the growth factor both transcriptionally and post-transcriptionally. Several studies demonstrated that an aberrant 14-3-3γ expression is associated with neurological disorders resulting from abnormalities in neuronal migration (3, 7, 9). Kittler et al. demonstrated that 24 h after Δ9-tetrahydrocannabinol treatment the 14-3-3γ subunit resulted upregulated in the rat hippocampus (10). Moreover, some authors reported that the *YWHAG* gene is involved in the MAPK pathway through the RAF-1 bond and the mTORC1 signaling pathway (11). Similarly to what occurs in other overactive-mTORC1-signaling-correlated disorders (TSC or FCD), we hypothesize that *YWHAG* gene haploinsufficiency can lead to an altered expression of CB1 receptors in neurons, which, in turn, influences the clinical response to CBD treatment positively. In line with this hypothesis, Hengstschlager et al. demonstrated an increase in 14-3-3γ levels (and other 14-3-3 proteins) when an ectopic overexpression of *TSC1* and *TSC2* occurs (the genes causing TSC) (12). Moreover, some studies reported that CB1 receptors seem to be linked to the activity of the 14-3-3 protein family. In particular, it has been proven that CB1 receptor activation can inhibit cell cycle progression by modulating 14-3-3β (13).

All these findings support the possibility of an altered expression of CB1 receptors that, probably, influence positively the CBD treatment (likely in TSC or FCD).

Herein, we firstly reported a patient with WBS and drug-resistant epilepsy, which was successfully treated with CBD; further extensive and controlled clinical studies are needed to verify our hypothesis and confirm the validity of CBD in these patients.

## Data Availability Statement

The original contributions presented in the study are included in the article/supplementary material, further inquiries can be directed to the corresponding author/s.

## Ethics Statement

Ethical review and approval was not required for the study on human participants in accordance with the local legislation and institutional requirements. Written informed consent to participate in this study was provided by the participants' legal guardian/next of kin. Written informed consent was obtained from the individual(s), and minor(s)' legal guardian/next of kin, for the publication of any potentially identifiable images or data included in this article.

## Author Contributions

MSp and AN conceived and planned the study. AN developed the theory and wrote the manuscript with support from MSa, GV, and AD. GD revised the project. All authors made substantial contributions to the critical review, editing, and revision of the final version of the manuscript.

## Conflict of Interest

The authors declare that this study received funding from GW Pharmaceuticals plc. London (London, United Kingdom). The funder was not involved in the study design, collection, analysis, interpretation of data, the writing of this article or the decision to submit it for publication.
